# Comparing different methods used to collect material for a microbiological evaluation of patients with chronic rhinosinusitis

**DOI:** 10.1590/S1808-86942010000300009

**Published:** 2015-10-20

**Authors:** Karina Mantovani, Daniela de Oliveira Rodrigues, Edwin Tamashiro, Fabiana Cardoso Pereira Valera, Ricardo Cassiano Demarco, Roberto Martinez, Wilma Terezinha Anselmo Lima

**Affiliations:** 1 MSc; Professor; 2 Resident physician; ENT; 3 PhD, Assistant Physician; 4 PhD, Professor; 5 MSC; Assistant Physician; 6 Associate Professor; 7 Associate Professor

**Keywords:** culture, microbiology, sinusitis

## Abstract

There is still controversy on which is the best method to collect the secretion directly from the middle meatus or maxillary sinus in patients with chronic rhinosinusitis.

**Aim:** To evaluate the prevalence of bacteria in patients with chronic rhinosinusitis and compare the suction trap collector to direct aspiration attached to a syringe for the microbiological analysis of these secretions.

**Materials and Methods:** Prospective study involving 31 patients who underwent endoscopically guided maxillary secretion aspiration by two different methods (aspiration with the collector tube “suction trap” and aspiration with the use of a catheter connected to a syringe), to determine the microbiological diagnosis and to compare the two methods used.

**Results:** microorganisms grew samples collected from 55% of the 31 patients. The most frequent bacteria were S. aureus, Pseudomonas aeruginosa and other aerobic Gram-negative bacteria. The results from cultures were similar between the two methods in 71% of patients.

**Conclusion:** S. aureus, Pseudomonas aeruginosa and other aerobic Gram-negative bacteria make up the main flora in the maxillary sinus of the patients. There was good correlation between the microbiological results obtained by using a catheter attached to a syringe and the “suction trap” nasal collector.

## INTRODUCTION

Despite the different studies done with Chronic Rhinosinusitis (CRS), we still do not know clearly the true pathogenic mechanisms and etiological agents participating in this disease. Contrary to the findings from microbiology studies carried out in patients with acute rhinosinusitis, there is no definitive and consistent data on the true distribution of bacterial pathogens present in patients with CRS. Part of this uncertainty is due to the major variability of the methods used in these studies (different collection methods, prior use of antibiotics, variations in culture methods), besides the difficulties in distinguishing which are the pathogens and which are only colonizing agents.

Maxillary sinus punction through the canine tooth fossa was until recently considered the gold standard method to collect samples for microbiological studies in patients with sinusitis. Nonetheless, it is a painful and invasive procedure, which depends on patient collaboration and sometimes requires local anesthesia, sedation or even general anesthesia^1^,^2^. Less invasive alternatives have been used, such as the collection of material from the middle meatus under endoscopic view. Collection under direct view with meatal swab or syringe aspiration can be criticized as to possible contamination from areas adjacent to the middle meatus, such as the nasal vestibule, which could yield not so reliable results with regards to the real microbiology of the paranasal sinus involved. Thus, more accurate techniques such as direct aspiration of secretion with a sterile container (“suction trap”) could solve such conflict. Studies comparing microbiological results obtained from the maxillary sinus punction with the endoscopic collection of material from the middle meatus have proven a good correlation between the methods3-6. Despite the broad acceptance of less invasive techniques, there is no evidence in regards of the equivalence between the different methods used to collect secretions from the middle meatus.

With this in mind, we compared the two methods used to collect material from the maxillary sinus: aspiration of nasal secretion with a sterile device (“suction trap”), and aspiration using the catheter with a syringe coupled to it, comparing the prevalence of pyogenic microorganisms between the two methods.

## MATERIALS AND METHODS

We carried out a cross-sectional study involving 31 consecutive patients – 12 males (38.7%) and 19 females (61.3%), between 13 and 78 years, (mean of 41.6 years), diagnosed with CRS. This project was previously approved by the Ethics in Research Committee – protocol # 1930/97).

We included patients with CRS who did not have clinical improvement after treatment (nasal saline flushing, topical and systemic steroids and antibiotics), who had indications of nasosinusal endoscopic surgery, according to the EPOS 2007 consensus^7^. We excluded the patients who had used antibiotics within 30 days prior to the sample collection, or those who had some anatomical alteration that prevented direct visualization of the middle meatus.

**Figure 1:**
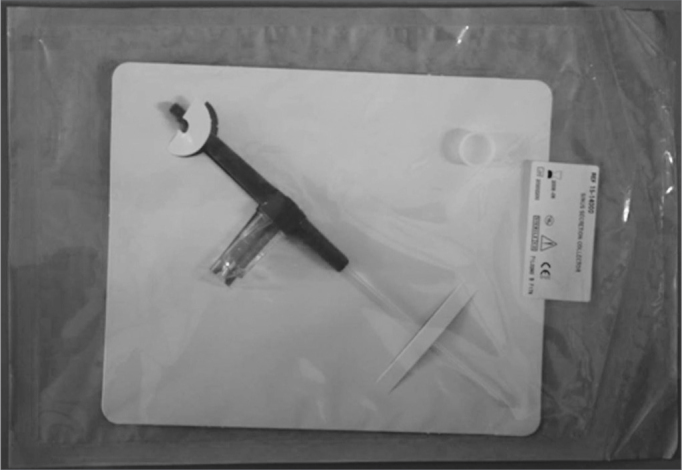
“suction trap”- sterile collector.

After anesthesia, the nasal cavity was submitted to a rigorous cleaning with saline solution and vasoconstriction with adrenalin 1:10000 during ten minutes. Afterwards, we accessed the maxillary sinus through nasal endoscopic surgery and material was collected from the maxillary sinus by means of different aspiration methods:

1. Catheter connected to a syringe, introduced all the way to the maxillary sinus;
2. Sterile collector “suction trap” (nasal secretion collection device – Medtronic Xomed, Jacksonvile/ FL -USA) (Figure 1).

The material collected was immediately seeded for aerobic bacteria study in blood Agar (Mueller Hinton Agar with 5% of goat blood), Mac Conkey (Agar, peptone, sodium chloride, biliary salts, Violet crystal, lactose and neutral red pH indicator) and Ni (simple Agar with 7.5% of NaCl) incubated at 37°C, during 24 hours. In order to identify the genus and species of the microorganisms isolated we used proper panels from the VITEK automated system®, adding the additional tests when necessary.

For the statistical analysis we used the Fisher's test in order to compare the positiveness of the samples, considering significant p<0.05.

## RESULTS

Of the 62 samples investigated (31 collected with a syringe and 31 using the nasal collector), (59.7%) microorganisms did not grow. Comparing the two collection methods, we found 20 negative samples obtained from a syringe (64.5%) and 17 negative samples obtained from the collector (54.8%). The Fisher test showed a p value of 0.60, showing that there was no significant difference between the two collection methods in relation to the number of samples which had bacterial growth.

Among the 25 samples which had microorganisms growing, 14 of them were obtained with the collector (one of the samples showed polymicrobial growth) and 11 obtained from a syringe. Comparing the microbiological profile between the two types of collection, we observed the growth of eleven Gram-negative bacteria and four Gram-positive in the syringe group, and *Staphylococcus aureus* was the most frequent in both groups (26.7% of the positive samples were obtained from the collector and 18.2% of the positive samples were obtained from the syringe). Other microorganisms we found were: *Pseudomonas aeruginosa, Streptococcus viridans, Enterobacter aerogenes, Staphylococcus epidermidis, Proteus mirabilis,* unidentified gram-negative bacilli, *Klebsiella pneumoniae, Enterobacter cloacae, Haemophilus sp and Escherichia coli* (Table 1).

**Table 1:** With syringe and catheter.

CASE	AGE	GENDER	WITH THE SUCTION TRAP			WITH SYRINGE AND CATHETER		
			Aerobic B.	Anaerobic B	Fungus	Aerobic B.	Anaerobic B	Fungus
1	13	F	–	–	–	–	–	–
2	47	F	–	–	–	*Streptococcus viridians*	–	–
3	58	F	–	–	–	–	–	–
4	25	M	*Enterobacter aerogenes*	–	–	*Enterobacter aerogenes*	–	–
5	72	F	*S. aureus*	–	–	–	–	–
6	22	F	–	–	–	*S. epidermidis*	–	–
7	51	M	*Proteus mirabilis*	–	–	–	–	–
8	45	M	*Bacilo Gram-negativo*	–	–	*Bacilo Gram-negativo*	–	–
9	49	M	*P. aeruginosa*	–	–	–	–	–
10	15	F	*P aeruginosa*	–	–	–	–	–
11	78	F	*Klebsiella pneumoniae*	–	–	–	–	–
12	43	F	–	–	–	–	–	–
13	46	M	*S. aureus + K. pneumoniae*	–	–	*Klebsiella pneumoniae*		
14	46	F	–	–	–	–	–	–
15	46	M	–	–	–	–	–	–
16	54	M	–	–	–	–	–	–
17	39	F	–	–	–	–	–	–
18	24	F	–	–	–	*Enterobacter cloacae*	–	–
19	23	F	–	–	–	–	–	–
20	27	F	*P. mirabilis*	–	–	–	–	–
21	55	M	*Haemophilus sp*	–	–	*Haemophilus sp*	–	–
22	50	M	–	–	–	–	–	–
23	70	F	*E. coli*	–	–	*E. coli*	–	–
24	28	M	*P. aeruginosa*	–	–	*P aeruginosa*	–	–
25	62	F	*S. aureus*	–	–	*S. aureus*	–	–
26	61	F	–	–	–	–	–	–
27	24	M	–	–	–	–	–	–
28	14	F	–	–	–	–	–	–
29	49	F	–	–	–	–	–	–
30	34	M	*S. aureus*	–	–	*S. aureus*	–	–
31	19	F	–	–	–	–	–	–

Considering the positive and negative samples obtained between the two means of collection, we found that 22 of the 31 samples (71%) had coinciding results.

## DISCUSSION

We still do not know the definitive treatment of CRS, since we are still no fully knowledgeable about the pathophysiological mechanisms nor the etiological agents involved in this disease. Among the different treatment modes proposed, empirical use of antibiotics is one of the main approaches used to treat this condition. Nonetheless, the high cost of the broad spectrum medication, the high rate of treatment failure and resistant microorganisms have required a better explanation as to the microorganisms which are truly involved in CRS.

In order to identify these microorganisms, many studies have been published in order to establish numerous variables, such as the ideal site to collect the material for culture, the best collection method, the type of material to be sent to the lab (mucosa or secretion), or the specific way to identify the microorganisms (culture or Polymerase Chain Reaction – PCR)^1^,^8^, ^9^, ^10^, ^11^, ^12^, ^13^.

In recent years, many studies have tried to validate the culture done from samples collected by means of middle meatus endoscopy, and the middle meatus aspiration was investigated and established by Jiang et al. (1993)^14^. A new study from the same authors which was carried out in 1998^4^ showed that mucosa samples were not better for pathogen identification when compared to the analysis of the nasal secretion.

Araújo et al. (2003)^15^ showed that 80% of the cultures collected by maxillary sinus punction showed growth of the same microorganisms. These same authors suggest that the culture done from the middle meatus endoscopy is a feasible alternative to the maxillary punction for it is not a non-invasive and effective method to identify pathogens involved in CRS.

A study held by Orobello et al. (1991)^5^ involving 39 pediatric patients showed a high correlation between the cultures of middle meatus secretion, compared to the secretion collected from the maxillary and ethmoid sinus secretions, and this correlated with 83% from the maxillary sinus and 80% from the ethmoid. Another study done by Ozcan et al. (2002)^16^ assessing 127 adult patients also showed a high correlation (91.2%) between the bacterial growth seen from the secretion collected from the middle meatus when compared to the material collected from the ethmoidal sinus. Having such data, the endoscopic aspiration has been considered an adequate way to obtain material to monitor patients with CRS and to guide antibiotic therapy, replacing the canine fossa punction of the maxillary sinus.

A study done by Tantilipikorn et al.^17^ showed no significant difference between obtaining material through aspiration or through swabs under endoscopic view. Nonetheless, the quantity of material obtained with the swabs is usually insufficient to look for fungi or anaerobic bacteria, which constrains the use of such method.

In the present study we used two different methods in order to collect secretion from the maxillary sinus under endoscopic view. The first we used a catheter coupled to a syringe which, although considerably less expensive and easy to obtain from any medical service, there would be a greater risk of contamination because of the greater difficulty in handling the catheter and the possible contact with the nasal vestibule. The second method, with the suction trap device, previously used to obtain material in other studies, is considered an effective method to avoid contamination, once the material is transferred by suction, from the paranasal sinus directly to a sterile container^6^,^18^, ^19^, ^20^, ^21^.

We found a significant number of samples without bacterial growth, with the collector (54.8%) and with a syringe (64.5%). Such results match those from previous studies which reported absence of bacterial growth in 17 to 60% of the samples^22^, ^23^, ^24^. Recent evidence suggest that the lack of bacterial growth in conventional cultures could be associated with the presence of bacterial biofilms adhered to the sinusal mucosa, or reinforcing the hypothesis that the inflammatory reaction found in cases of CRS is not always associated with infectious processes^25^.

The most frequent microorganisms found in the samples collected were similar to those from previous studies: *Staphylococcus aureus* and *Pseudomonas aeruginosa.* Nonetheless, we found a higher number of gram-negative aerobic bacteria than was reported in other studies^2^,^15^,^26^,^27^. Comparing the samples collected with the syringe with those collected with the suction trap, we observed the same results in 71% of the patients. In the eight patients in whom there were microorganisms of both types isolated in the samples, there was 100% match as to the species of bacteria, except for the additional detection of *Staphylococcus aureus* in a sample obtained from a smaller collector. Even seeing this difference in the results, as per previously reported, there was no statistically significant difference regarding the use of these two methods (p = 0.60).

Among disagreeing results, we noticed that among the patients in whom only positive cultures were seen with the use of the suction trap, there was also the growth of *Staphylococcus aureus, Proteus mirabilis, Pseudomonas aeruginosa* and *Klebsiella pneumoniae.* The lack of growth of these pathogens in the corresponding analysis collected with the syringe shows a probable false-negative result.

Among patients who had positive cultures with the syringe only, we found the growth of Streptococcus viridans, *Staphylococcus epidermidis,* and *Enterobacter cloacae.* The first two bacteria are considered by some studies as colonizers of the nasal cavity^18^,^19^,^27^.

Therefore, although the statistical analysis did not show differences between these two methods, we cannot rule out the fact that collecting material with the trap provides more specific results than the use of a catheter coupled to a syringe, since the statistical analysis does not consider which microorganisms were found in the culture.

Future studies, with larger number of patients, better standardization of collection methods and other more specific analysis done in the maxillary sinus secretion, as microbiological evaluation by PCR and assessment of the inflammatory cells infiltrate present in the nasal mucosal samples, would be necessary to confirm whether the use of the suction trap would truly be a more accurate method for the microbiological diagnosis of patients with CRS.

## CONCLUSION

In the present study we found a predominance of *Staphylococcus aureus, Pseudomonas aeruginosa* and other Gram-negative bacteria among the pathogens associated with CRS. We have also noticed that the catheter-coupled-to-the-syringe method present similar results to those obtained with the suction trap, and it can be a valid and reliable method to obtain secretion from the middle meatus, provided the proper care is taken in order to avoid contamination from the nasal vestibule.
